# Transglutaminase-2 Mediates the Biomechanical Properties of the Colorectal Cancer Tissue Microenvironment that Contribute to Disease Progression

**DOI:** 10.3390/cancers11050701

**Published:** 2019-05-21

**Authors:** Robin Delaine-Smith, Nicola Wright, Chris Hanley, Rebecca Hanwell, Rahul Bhome, Marc Bullock, Cole Drifka, Kevin Eliceiri, Gareth Thomas, Martin Knight, Alex Mirnezami, Nicholas Peake

**Affiliations:** 1 School of Engineering and Materials Science, Queen Mary University of London, London E1 4NS, UK; r.delaine-smith@qmul.ac.uk (R.D.-S.); m.m.knight@qmul.ac.uk (M.K.); 2 Biomolecular Research Centre, Sheffield Hallam University, Howard Street, Sheffield S1 1WB, UK; nicolawrightx@yahoo.co.uk (N.W.); rebeccahanwell@sky.com (R.H.); 3 Cancer Sciences Unit, Faculty of Medicine, University of Southampton, Tremona Road, Southampton SO16 6YD, UK; c.j.hanley@soton.ac.uk (C.H.); rahulbhome@doctors.org.uk (R.B.); m.bullock@soton.ac.uk (M.B.); g.thomas@soton.ac.uk (G.T.); a.h.mirnezami@soton.ac.uk (A.M.); 4 Department of Surgery, Southampton University Hospital NHS Trust, Southampton SO16 6YD, UK; 5 Laboratory for Optical and Computational Instrumentation, University of Wisconsin at Madison, Madison, WI 53706, USA; cdrifka@umn.edu (C.D.); eliceiri@wisc.edu (K.E.)

**Keywords:** colorectal, transglutaminase, remodelling, biomechanics, collagen, extracellular matrix, tumor microenvironment

## Abstract

Colorectal cancer is the third most common cancer worldwide, and the fourth leading cause of malignancy-related mortality. This highlights the need to understand the processes driving this disease in order to develop new treatments and improve patient outcomes. A potential therapeutic target is the increased stiffness of the tumour microenvironment, which is linked to aggressive cancer cell behaviour by enhancing biomechanical signalling. In this study, we used an siRNA-based approach to investigate the contribution of the protein cross-linking enzyme transglutaminase-2 (TG2) to matrix remodelling and biomechanical properties of the tumour microenvironment. TG2 inhibited cancer cell growth in organotypic 3D fibroblast/SW480 co-culture models, and biomechanical analysis demonstrated that colorectal cancer cells induced fibroblast-mediated stiffness which was inhibited by silencing TG2. These biomechanical changes were associated with observed alterations to collagen fibre structure, notably fibre thickness. Our in vitro findings of collagen composition changes were also seen with imaging biopsied tissues from patients with colorectal cancer, with TG2 correlating positively with thicker collagen fibres, and associating with poor outcome as determined by disease recurrence post-surgery and overall survival. In conclusion, this study demonstrates a role for TG2 in the stromal response to invading tumour, leading to tissue stiffening and poor outcome in patients.

## 1. Introduction

Colorectal cancer (CRC) is the third most common cancer worldwide, and the fourth leading cause of malignancy related mortality [1]. When detected and treated early, prognosis is good, with 5-year survival rates of 90% for patients with grade I disease. However, despite improvements in screening, around 50% of patients are diagnosed with later stage disease where outcome is poor; 5-year survival rates fall to 25% for grade III and <10% for grade IV (metastatic) disease [2]. This highlights the importance of understanding the events that allow CRC to establish and progress, and characterising the processes linked to poor survival is therefore a priority.

The protein cross-linking enzyme transglutaminase-2 (TG2) has been linked to several cancer types and has been implicated in a variety of processes associated with cancer cell behaviour including chemoresistance, apoptosis, invasion, migration, stemness and involvement in epithelial-mesenchymal transition [3,4,5,6,7,8,9]. The primary function of TG2 is the formation of cross-links between glutamine and lysine residues of target proteins, and a diverse range of substrates and downstream mediators have been identified which are linked to the functional effects of TG2. These include critical intracellular proteins such as PTEN [10], actin [11] and NF-kB [3], cell surface components such as integrins [7,12] and components of the focal adhesion kinase (FAK) complex [7,13], and extracellular matrix (ECM) components such as collagen and fibronectin [12,14]. The wide-ranging and context-dependent effects of TG2 therefore appear linked to specific intra- and extra-cellular localisation and availability of specific substrates [5,15].

We have shown that epithelial TG2 expression is observed in early stages of CRC, but then appears to be down-regulated as tumours progress towards more invasive, later stages of disease [6]. This loss of TG2 in late stage disease appeared to enhance invasive behaviour of CRC cells. This previous work led us to propose that TG2 may play an important role in CRC development and progression in early stages of disease, but that there may be a selective advantage for CRC cells to down-regulate TG2 in later-stage disease to facilitate invasion and metastasis. We also noted that TG2 expression is prominent in tumour-associated stroma [6]. Given the capability of extracellular TG2 to react with components of the ECM, we and others have proposed that in some contexts, TG2 acts to restrict invasion [6,16]. Previously, it has also been shown that cross-linking and subsequent stiffening of the ECM can lead to increased invasion of cancer cells [17,18]. Much of this work has focused on the involvement of the cross-linking enzyme Lysyl Oxidase (LOX) in promoting matrix stiffness, enhancing biomechanical signalling and subsequent aggression [19]; however, recent studies have logically associated TG2 with these events, proposing that this may occur through interactions between cancer cells and fibroblasts associated with the tumour site [20].

Cancer-associated fibroblasts (CAFs) localised within the stroma adjacent to an invasive tumour are known to play a key role in progression of the disease, and are the primary drivers of collagen synthesis and remodelling observed in the highly desmoplastic environment found in CRC [13,21]. Cross-talk between CAFs and the invading CRC cells involving key mediators such as Transforming Growth Factor (TGF)-β plays an important role in shaping the progression of disease [22,23,24], and there is a close relationship between TGF-β and TG2 [25,26]. Based on the observations of ourselves and others; this indicates that TG2 has pleiotropic and context-dependent activities during the development and progression of CRC. We hypothesise that cancer-derived TG2 may promote cancer cell survival early in disease, with stromal, fibroblast-derived TG2 functioning to restrict tumour growth through strengthening of the ECM. As disease progresses, stiffening of the ECM may drive progression towards a more aggressive and invasive phenotype through increased biomechanical signalling. Therefore, the objectives of this study were to examine the relationship between TG2 and the biomechanical environment through modification of the ECM, to understand the differential impact of epithelial and stromal TG2 using specific TG2 knockdown approaches in an in vitro system, and to link these functional observations to imaging-based fibre alteration measurements in the collagen rich ECM observed in patients with CRC.

## 2. Results

### 2.1. Fibroblasts Differentially Mediate Growth of SW480 Cells in an In Vitro Model of CRC

**Figure 1:**
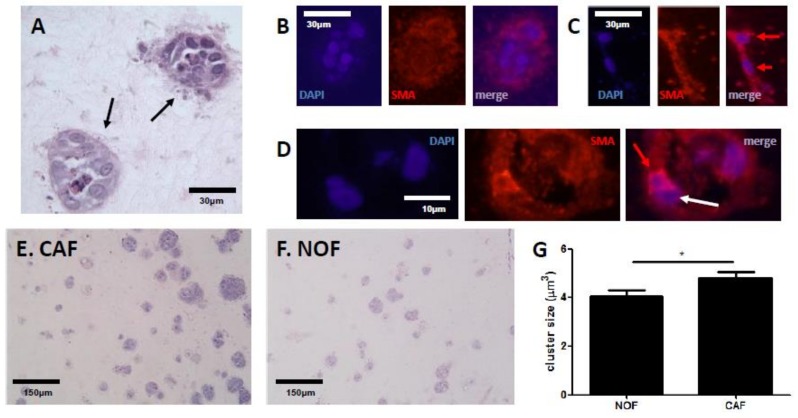
Colon fibroblasts mediate SW480 growth in a co-culture 3D model. Co-culture of fibroblasts with SW480 cells (**A**, H&E stain), with clearly defined invasive front and stromal boundary as well as small particles that appear to be released from the clusters (black arrows). Little SMA staining was observed in the clusters (**B**), but fibroblasts were observed in the surrounding regions, with NOFs demonstrating SMA expression (**C**, individual cells indicated by red arrows). By comparison CAFs showed higher expression levels of SMA (**D**, SMA-positive cell indicated by red arrow), and interacted with SW480 cells (indicated by white arrow). Cluster growth was greater in co-cultures with CAFs (**E**) compared to NOFs (**F**); quantification of SW480 cluster sizes showed significantly higher sizes in co-culture with CAFs compared to NOFs (**G**, *n* = 179 NOF, 303 CAF). * *p* < 0.05.

TG2 is clearly expressed at the CRC/stromal boundary in patients [6], and is expressed by CAFs [27]. We therefore established 3D cultures of colon fibroblasts in order to develop a model that would allow examination of ECM remodelling and biomechanical changes linked to TG2 as a consequence of SW480/colon fibroblast interaction using collagen as a physiological TG2 ECM substrate. Using a series of paired cancer-associated fibroblasts (CAFs) and normal fibroblasts (NOFs), the CRC cell line SW480 was then introduced into the 3D cultures, and co-cultures maintained for 7 days before analysis. Co-culture of SW480 cells with colon fibroblasts resulted in clear, distinct cancer clusters consisting of between 5–20 cells (Figure 1A). Fibroblasts within the model were also observed around the perimeter of the clusters identified by SMA staining while there was little SMA within the clusters (Figure 1B). SMA-positive cells were identified both in cultures with NOFs and CAFs; NOFs appeared as elongated, thin cells and were observed associating with each other, aligning end-to-end (Figure 1C), whereas CAFs had higher expression of SMA, tended to be less elongated, and were observed to associate with SW480 cells (Figure 1D). The role of fibroblasts in mediating CRC growth was next established in the 3D co-culture model, by comparing the relative size of SW480 clusters in co-cultures with CAFs or NOFs. We observed that overall, SW480 cells cultured with CAFs had increased growth of the clusters (Figure 1E) when compared to SW480 cells cultured with NOFs (Figure 1F), and this was statistically significant (Figure 1G).

### 2.2. TG2 Activity Is Prominent at the CRC/Stroma Boundary in Co-Culture Models

**Figure 2:**
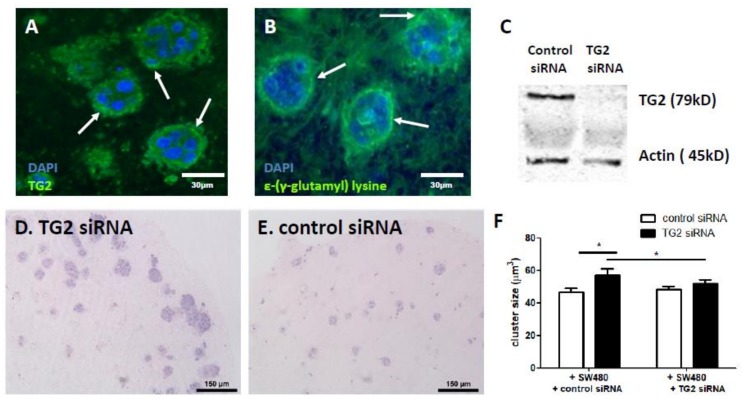
TG2 at the SW480 cluster boundary mediates CRC growth. TG2 is prominently expressed at the invasive edge of SW480 clusters (**A**, white arrows indicating CRC/stroma boundary). TG2 activity in the co-cultures was observed by staining for the ε-(γ-glutamyl) lysine isopeptide cross-link (**B**), and prominent at the invasive edge (white arrows indicating CRC/stroma boundary). TG2 siRNA was delivered to fibroblasts (**C**), and co-culture with TG2-silenced fibroblasts resulted in greater cluster growth (**D**,**E**). Quantification of cluster size indicated that this increase was statistically significant (**F**, *n* = 27). TG2 siRNA delivered to SW480 cells blocked the enhanced cluster size induced by TG2 siRNA delivered to fibroblasts (**F**, *n* = 27). * *p* < 0.05.

SW480 cells express relatively high levels of TG2 in comparison to SW620 cells (Supplementary Figure S1A), and the level of TG2 influences the invasive behaviour of these cells [6]. Silencing TG2 in SW480 cells led to SW480-only gels consisting of thicker, sparser collagen fibres (Supplementary Figure S1B,C), and gels increased in stiffness as indicated by modulus (Supplementary Figure S1D). In co-cultures, prominent expression of TG2 at the invasive front was observed (Figure 2A), providing a useful model of the CRC/stroma boundary and suggesting that fibroblast activation through cross-talk with CRC cells is critical for TG2 expression in the tumour microenvironment (TME). Moreover, evidence for TG2-mediated cross-linking in the TME was observed by staining for the ε-(γ-glutamyl) lysine isopeptide link catalysed by TG2, which was observed throughout the co-cultures (Figure 2B) and prominently at the CRC/stroma boundary.

### 2.3. Fibroblast-Derived TG2 and CRC-Derived TG2 Have Differential Effects in the TME

To assess the contribution of TG2 to CRC growth, we silenced TG2 in fibroblasts (Figure 2C) and saw significantly enhanced growth of SW480 clusters when compared to unsilenced controls (Figure 2D–F), pointing to fibroblast TG2 as a mechanism restricting cluster size. Since both fibroblast and CRC components of the co-cultures express TG2, we then compared the impact of silencing TG2 in fibroblasts with that of silencing TG2 in SW480 cells. Interestingly, silencing TG2 expression in the SW480 cells resulted in a loss of the enhanced cluster growth promoted by silencing TG2 in the fibroblasts (Figure 2F), suggesting that CRC-derived TG2 contributes to driving growth of the clusters, in a mechanism that then involves fibroblast-derived TG2. Silencing TG2 in either fibroblasts or SW480 cells had no impact on the number of clusters that established (Supplementary Figure S2A).

TG2 is known to mediate upregulation of NF-kB and subsequent release of cytokines such as IL-6 and IL-8 [3,6]; we therefore also assessed cytokine profiles in the co-cultures. IL-8 release was significantly lower in co-cultures with CAFs compared to NOFs (Supplementary Figure S2B), and co-culture with SW480 cells further inhibited IL-8 release (Supplementary Figure S2C). Notably, silencing TG2 in fibroblasts inhibited the down-regulation of IL-8 induced by SW480 cells, again demonstrating CRC-driven, TG2-mediated cross-talk between the cells in co-culture. IL-6 release was not significantly different, although the overall trend was similar to that observed for IL-8 (Supplementary Figure S2D).

### 2.4. TG2 Mediates the Biomechanical Properties of 3D Co-Culture Gels

**Figure 3:**
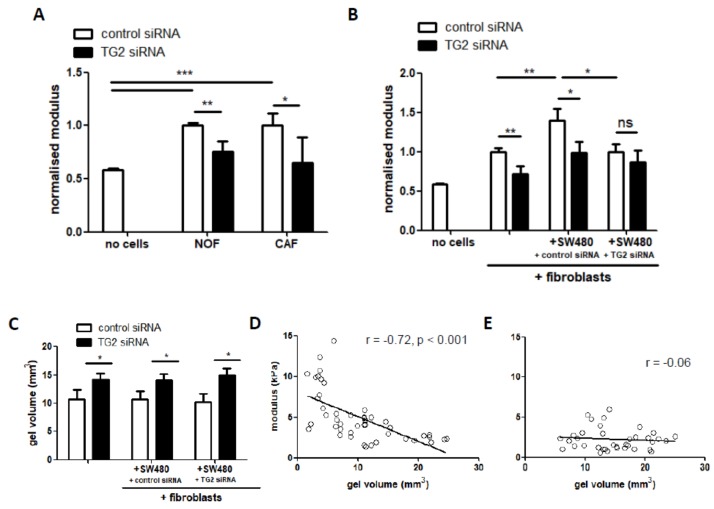
TG2-mediated changes alter the biomechanical properties of CRC/fibroblast co-cultures. Incorporation of fibroblasts into collagen gels significantly increased modulus compared to blank gels (**A**, *n* = 8–11). Silencing of TG2 caused a significant decrease in modulus but no differences between NOFs and CAFs. Co-culture of fibroblasts with SW480 cells resulted in a significant increase in modulus (**B**, *n* = 9–11); however, silencing of TG2 in SW480 cells blocked this increase. Silencing of TG2 in fibroblasts also resulted in a loss of contractile ability (**C**, *n* = 19). A clear correlation between gel volume and modulus was observed in gels incorporating fibroblasts (**D**), but this relationship was lost following silencing of TG2 in the fibroblasts (**E**). * *p* < 0.05, ** *p* < 0.01, *** *p* < 0.001, ns = not significant.

To understand how TG2-mediated structural alterations affect mechanical properties, the gel modulus was measured following manipulation of TG2 in either the fibroblasts and/or the SW480 cells. In monocultures, both NOF and CAF lines significantly increased modulus compared to cell-free gels (Figure 3A). No significant differences in modulus were observed between gels containing NOFs or CAFs; however, modulus was significantly decreased after silencing TG2 in both fibroblast types (Figure 3A). Next, we examined the impact of co-culturing SW480 cells with the fibroblasts. Co-culture significantly increased the modulus compared to gels with fibroblasts only (Figure 3B), and silencing fibroblast-derived TG2 in the co-culture gels led to decreased modulus. Interestingly, silencing TG2 in the SW480 cells also led to a decrease in modulus (Figure 3B)—manipulating TG2 in fibroblasts did not have any effect once SW480-derived TG2 was silenced. These data suggest that the fibroblast-derived TG2 contributes to changes in the biomechanical environment and that these changes are influenced by CRC-derived TG2.

The relationship between TG2 and contractile forces acting on the cultures was assessed by measuring gel volume in the co-culture gels and comparing treatments with and without silencing of TG2. As expected, fibroblasts caused significant gel contraction over 7 days. Gels contracted by around 8-fold with the addition of fibroblasts only, and silencing TG2 resulted in a significant 1.3-fold reduction in fibroblast contraction of the gels (Figure 3C). Addition of SW480 cells with or without TG2 siRNA in co-cultures did not significantly impact gel volume (Figure 3C). Analysis of all co-cultures with untreated fibroblasts indicated a significant inverse correlation between volume and modulus (Figure 3D). However, in gels with silencing of fibroblast TG2, a loss of coupling between contractility/volume and modulus was observed (Figure 3E). SW480-derived TG2 did not significantly alter the relationship between volume and modulus (Supplementary Figure S3A–C), although the inverse correlation was strongest when TG2 was silenced in the SW480 cells (Supplementary Figure S3C).

### 2.5. TG2 Influences Collagen Matrix Properties in Fibroblast/SW480 Co-Cultures

**Figure 4:**
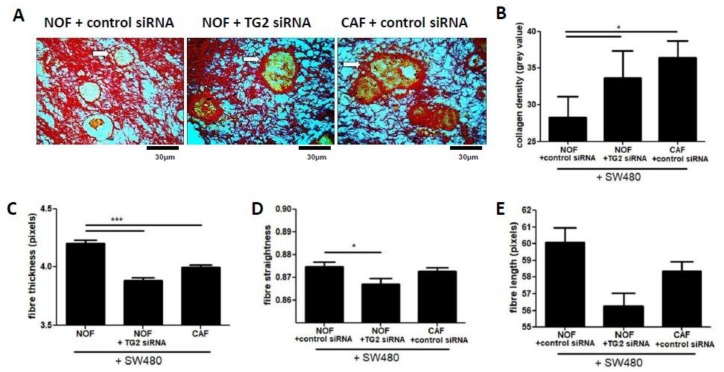
TG2 mediates structural features of collagen fibroblast/CRC co-cultures. Sirius red staining of fibroblast/SW480 co-cultures showed distinctive patterns of dense collagen accumulation at the CRC/stroma boundary (**A**, white arrows). Quantification of the collagen density at this boundary indicated that treating NOFs with TG2 siRNA caused a significant increase of collagen density in these areas (**B**, *n* = 10–17). However, treating NOFs with TG2 siRNA lead to overall lower collagen fibre thickness in the gels (**C**, *n* =10–17) and fibres that were significantly less straight (**D**, *n* = 10–17). No difference in fibre length was observed between the conditions (**E**, *n* = 10–17), despite a trend towards shorter fibre length in gels cast with NOFs treated with TG2 siRNA (**E**). * *p* < 0.05, *** *p* < 0.001.

To establish the structural basis for the biomechanical changes mediated by TG2 in the co-culture model, we performed analysis of collagen structure using quantitative analysis of sirius red-stained gel sections. The SW480 cell clusters were clearly defined by a dense accumulation of collagen at the invasive edges, set within a background collagen matrix of diffuse, fine fibres (Figure 4A). Quantification of staining intensity of the boundary regions showed that collagen density was significantly higher around CRC clusters co-cultured with CAFs compared to NOFs (Figure 4A,B), and silencing TG2 in NOFs induced a similar increase in density compared to CAFs (Figure 4B). We next quantified structural features of the collagen fibres throughout the stained co-culture gels using curvelet-transform fibre extraction (CT-FIRE [28]). This analysis indicated that co-cultures with CAFs had significantly lower overall collagen fibre thickness compared to cultures with NOFs, and similarly silencing TG2 in NOFs resulted in significantly reduced fibre thickness (Figure 4C). We also observed that silencing TG2 in NOFs resulted in a significant decrease in straightness of the fibres, although this observation was not mirrored in co-cultures with CAFs (Figure 4D). Co-cultures with both NOFs treated with TG2 siRNA and with CAFs resulted in shorter fibres than co-cultures with NOFs (Figure 4E), although these differences were not statistically significant. Overall, these data indicated that loss of TG2 resulted in lower overall collagen fibre thickness throughout the gel, implying a role for TG2 in fibre thickening mediated by fibroblasts. However, silencing TG2 also increased the density of fibres at the CRC/stroma boundary, which may indicate compressive forces at the boundary of the expanding clusters.

### 2.6. SW480-Derived TG2 Drives Alterations to Collagen Fibre Structure that Are Mediated by Fibroblasts

**Figure 5:**
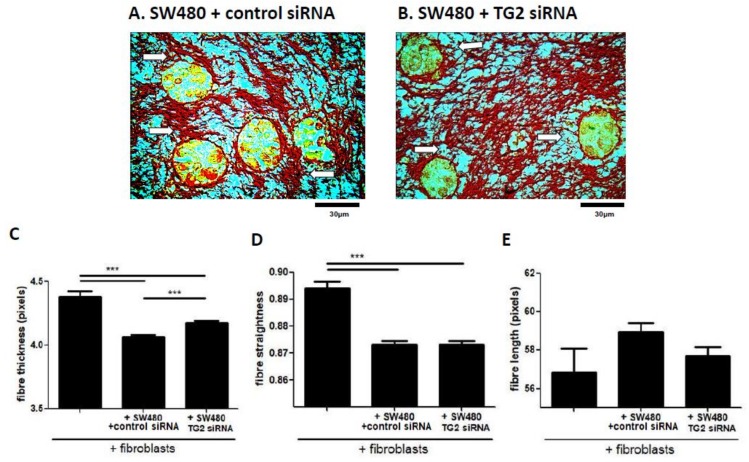
SW480-derived TG2 drives fibroblast-mediated structural alterations in co-cultures. Sirius red staining of fibroblast/SW480 co-cultures showed regions of dense collagen at the CRC/stroma boundary with untreated SW480 cells (**A**, white arrows) and thinning regions at this boundary once SW480 cells were treated with TG2 siRNA (**B**, white arrows). Collagen fibre thickness was significantly lower once SW480 cells were introduced into cultures, compared to gels cast with fibroblasts alone (**C**, *n* = 27). When treated with TG2 siRNA, the reduced fibre thickness induced by SW480 cells was partially reversed (**C**, *n* = 27). SW480 cells also resulted in less straight fibres compared to gels cast with fibroblasts alone (**D**, *n* = 27), but TG2 siRNA treatment of the SW480 cells had no effect. Significant differences in fibre length were not observed between gels cast with fibroblasts alone, SW480 cells, or with SW480 cells treated with TG2 siRNA (**E**, *n* =27). *** *p* < 0.001.

Having assessed the alterations in the collagen structure of 3D co-cultures in response to fibroblast TG2, we next assessed the impact of CRC-derived TG2 using SW480 cells, with and without TG2 siRNA. Sirius red staining of SW480 co-cultures showed large clusters with areas of dense collagen surrounding the clusters (Figure 5A), compared with smaller clusters within a less diffuse, more homogenous collagen structure in co-cultures with TG2-silenced SW480 cells (Figure 5B). Fibre analysis demonstrated that SW480 cells caused a loss of fibre thickness in co-cultures compared to gels cast only with fibroblasts (Figure 5C). However, treating SW480 cells with TG2 siRNA partially inhibited this loss. SW480 cells also lead to significantly less straight fibres, but treating SW480 cells with TG2 siRNA had no impact (Figure 5D). No differences were observed between conditions in terms of collagen fibre length (Figure 5E). Overall, this data indicated that CRC cells drive thinning of collagen fibres, and that this process may involve CRC-derived TG2.

### 2.7. TG2 Regulates Fibroblast Lysyl Oxidase (LOX) Expression In Vitro

**Figure 6:**
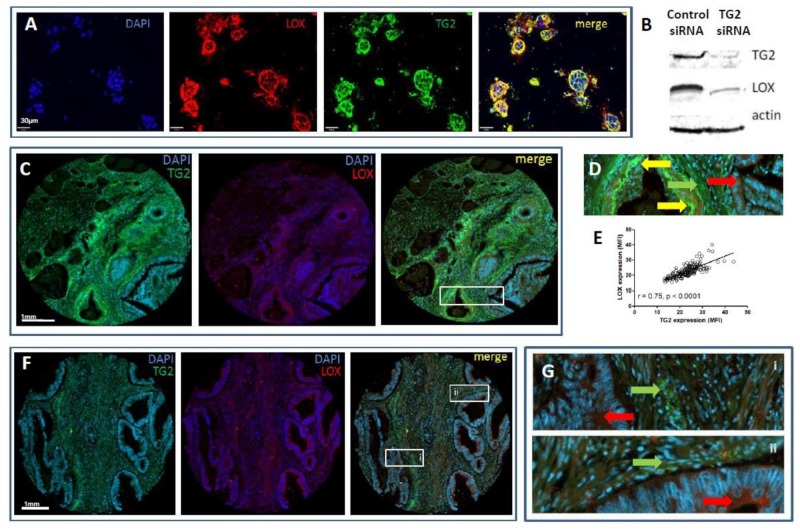
TG2 regulates LOX in vitro, but the enzymes have distinct expression profiles in vivo. Expression of LOX was observed at the CRC/stroma boundary of SW480/fibroblast co-cultures (**A**, red staining), which significantly co-localised with TG2 (green staining, co-localisation indicated by yellow colouration, right-hand panel). Inhibiting TG2 reduced the expression of LOX in cell lysates (**B**). To compare in vitro staining with patient tissues, a TMA was stained for both enzymes. Some co-localisation was observed (**C**, yellow arrows), primarily in stromal regions (**D**, magnified image of the boxed area in **C**); however TG2 was observed independent of LOX in other regions of stroma (green arrows), and LOX independent of TG2 in epithelial areas (red arrows). Overall mean tissue expression of TG2 and LOX was highly correlated (**E**), which was primarily due to closely expressed, but not co-localised, staining in the tissue stroma (**F**, and magnified images of boxes i and ii, **G**).

The involvement of LOX in the remodelling of stromal collagen and subsequent changes to the biomechanical properties of the tumour microenvironment has been well established [19]. Therefore, we assessed the relationship between these two cross-linking enzymes, TG2 and LOX. Immunostaining in the co-culture models showed a high degree of co-localisation (Figure 6A, Supplementary Figure S4A), notably at the CRC/stroma boundary. Quantitation of protein expression indicated a statistically significantly lower basal level of TG2 in gels seeded with CAFs compared to NOFs, but higher expression induced at this boundary (Supplementary Figure S4B). A similar pattern was observed for LOX expression, but the lower basal expression was not statistically significant (Supplementary Figure S4C). Furthermore, our siRNA experiments demonstrated that LOX expression was inhibited when fibroblasts were treated with TG2 siRNA, indicating a regulatory link between TG2 and LOX (Figure 6B).

### 2.8. Expression Profiles of TG2 and LOX Overlap, but Are Distinct in CRC Patients

We have previously demonstrated epithelial staining of TG2 in CRC tissue sections that is inversely correlated with invasive and metastatic features, as well as extensive staining in CRC stromal tissue [6]. We therefore assessed the expression of TG2 and LOX in tissues to assess whether their co-expression was reflected in patients, using a TMA that has been previously characterised [29]. Both TG2 and LOX were expressed in the cores (Figure 6C), and both epithelial and stromal staining was observed. In some tissues, co-localisation of the two enzymes were observed (Figure 6D), and quantification of mean expression per section demonstrated a correlation between overall expression of TG2 and LOX (Figure 6E). However, we also noted distinct staining patterns in the tissues, particularly extensive TG2 expression in tumour stroma, and LOX expression that was prominent in the epithelia (Figure 6F,G), indicating distinct regulation of the two enzymes in vivo.

### 2.9. TG2 Expression Correlates with Collagen Fibre Thickness in CRC Tissues

**Figure 7:**
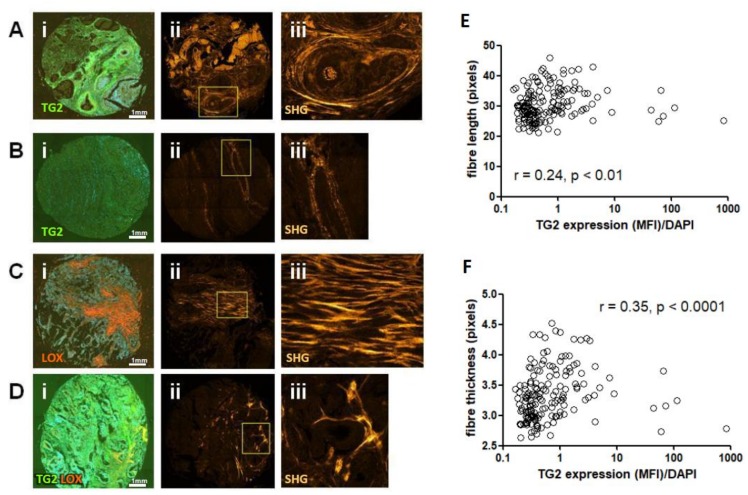
TG2, LOX and co-localisation of both correlate with collagen structural parameters determined by SHG. High TG2 expression (**A**, green staining in panel i) was associated with regions of thicker collagen fibres which surround the epithelium (**A**, panel ii and magnified image panel iii). By contrast, low TG2 expression (**B**, panel ii) was associated with regions of thinner fibres (panel ii and magnified image panel iii). High LOX staining (**C**, red staining in panel i) associated with thick fibres extending extensively throughout the stroma (panel ii and magnified image panel iii). Areas of high co-localisation of both enzymes (**D**, panel i, yellow colouration) showed distinctive, localised, thickened fibre bundles (**D**, panel ii and magnified image panel iii). Correlation analysis of staining and SHG imaging showed a significant relationship between TG2 and fibre length (**E**), and fibre width (**F**).

The CRC TMA used in this study has previously been analysed using with the collagen specific, label free method of second harmonic generation (SHG) microscopy [30]. This approach enabled us to next investigate whether the expression of TG2 and LOX were correlated with structural features of collagen in human patients. Using representative images from both immunofluorescence (IMF) staining (TG2 and LOX) and SHG analysis (fibrillar collagen), we observed that high TG2 expression in patients correlated with thicker, aligned collagen fibres surrounding the epithelium (Figure 7A), whereas low TG2 expression in patients correlates with thin fibres around the epithelium (Figure 7B). Where high expression of LOX was observed, thick fibres extending throughout the stroma were observed with little association with the epithelia (Figure 7C).

Co-localisation of TG2 and LOX expression was observed in distinct areas of shorter, individual, poorly aligned fibres (Figure 7D). The SHG data was next quantitively analysed for fibre thickness, straightness and length, and correlation analysis performed to link these variables to expression of TG2 and LOX. TG2 showed a weak but still significant correlation to collagen fibre length (Figure 7E), and a stronger, more significant correlation with fibre thickness (Figure 7F). To gain a better measurement of the co-localised TG2 and LOX signals, we also calculated Mander’s co-localisation coefficient (tM1) on the sections, which correlated weakly but significantly with fibre length (Supplementary Figure S4D). Overall, our analysis of 198 cores from CRC patients suggested that TG2 is linked to collagen fibre thickness, which mechanistically supports the observations in our in vitro 3D model illustrating a role for TG2 in tissue characteristics.

### 2.10. TG2 Expression Is Linked to Poorer Outcome in CRC Patients

**Figure 8:**
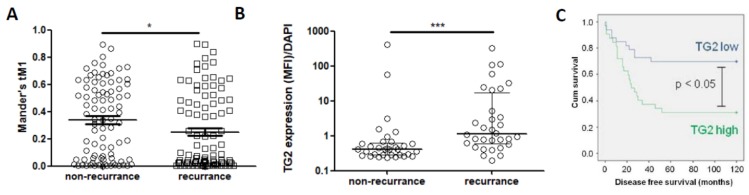
TG2 associates with poorer prognosis in CRC patients. Co-localisation of TG2 and LOX was significantly lower in patients with recurrence of disease (**A**). However, TG2 expression alone was significantly higher in patients with recurrence of disease (**B**), and high expression of TG2 was significantly associated with lower 5-year survival rates (**C**). * *p* < 0.05, ** *p* < 0.01.

To link the data on TG2, fibre analysis and CRC growth to patient outcome, the TMA quantification data was used to assess how expression levels linked to recurrence of disease post-surgery. Co-localisation of TG2 and LOX using tM1 was significantly lower in patients that went on to have disease recurrence (Figure 8A). However, when assessing TG2 alone, expression was significantly higher in patients that went on to have recurrence (Figure 8B). Since recurrence is associated with a poorer outcome, we therefore performed survival analysis on the TMA data. High TG2 levels were significantly associated with lower survival (Figure 8C).

## 3. Discussion

It is well established that extensive collagen deposition and remodelling occurs in the CRC tumour microenvironment, and that these processes can have an impact on disease progression. Interaction of the invading tumour with surrounding fibroblasts leads to collagen synthesis, with increased density of the collagen matrix adjacent to a tumour appearing to play an important defensive role in slowing invasion [21]. We have previously observed that the protein cross-linking enzyme TG2 is prominently expressed at the cancer/stroma boundary in CRC [6], and TG2 cross-linking of the ECM is known to slow invasive behaviour of cancer cells [16], pointing to a role for this enzyme in supporting the desmoplasia and enhanced collagen synthesis in response to invading tumours. However, these processes result in a stiffer ECM, and enhanced biomechanical signalling in response to increasing tissue stiffness can induce aggressive behaviour in cancer cells [17,18], Accordingly observations made by other groups have linked enhanced stromal TG2 to poor prognosis in some cancers, including CRC [31,32], and our study supports the observation that TG2 expression is linked to poorer outcome in terms of tumour recurrence and overall survival in CRC [32]. To better understand the relationship between TG2, cross-linking, ECM structure and cancer progression, we used a collagen gel culture system, embedding colon-derived fibroblasts in co-culture with CRC cells. This system was designed to allow siRNA-mediated manipulation of TG2 in both CRC and fibroblasts, and assessment of its functional role through biomechanical analysis and quantitative imaging of structural changes in the collagen matrix.

Reciprocal signalling between cancer cells and adjacent fibroblasts leads to differentiation towards a myofibroblast-like, contractile phenotype, characterised by expression of smooth muscle actin (SMA), and collagen remodelling at the tumour site [21,23,24]. In this study, we have demonstrated an important role for fibroblast-derived TG2 in mediating the biomechanical properties of the ECM. Fibroblast-derived TG2 causes ECM stiffening, and TG2 loss results in softer gels. Stiffness increases when fibroblasts are exposed to CRC cells, which could be a defensive response to an invading tumour. Guided by this data showing that TG2 impacts on biomechanical properties, and given the ability of TG2 to cross-link collagen, we proposed that TG2-mediated alterations to collagen structure were likely to underpin the biomechanical effects observed. Capturing collagen structure using sirius red staining, we utilised the fibre quantification tool CT-FIRE [28] to understand how TG2 contributes to collagen structure, analysing three variables: fibre thickness, fibre length and fibre straightness. Fibre length has recently been linked to poor prognosis in various cancer types [30], and the formation of a matrix consisting of radially aligned fibres is proposed to contribute towards invasive behaviour [33]. In this study, we established that TG2 was linked to collagen fibre thickness-silencing TG2 in fibroblasts reduced fibre thickness. This appeared to be clinically relevant, with TG2 expression correlating with fibre thickness in analysis of tissue sections from patients. The formation of thicker collagen fibres was associated with stiffer gels, providing a link between TG2, tissue structure and biomechanics. Furthermore, as with the biomechanical data, fibre thickening appeared to be induced by CRC-derived TG2, and inhibiting this CRC-derived signalling restored both fibre thickness and biomechanical properties.

The clearest evidence of a dense, desmoplastic response in our in vitro analysis appeared at the perimeter of the CRC clusters (Figure 4A,B). This region is also where significant TG2 activity was observed, along with significant co-localised LOX activity (Figure 6A) and a significant increase in collagen density (Figure 4B). While collagen density has been linked directly to cancer progression [34], thick, dense fibres at the cluster perimeter are also associated with better outcome by providing resistance to invasion [35]—which reflects our observations in vitro. Analysis of the collagen structure using SHG demonstrates that tissue sections with high TG2 show areas of thickened fibres surrounding the epithelium, with features similar to the TACS-2 morphology described for breast cancer tissues [34]—with features of stromal “stretching” and compressive restraint of the invading tumour. However, these regions were denser once TG2 expression was inhibited, suggesting that this dense accumulation is at least partially a consequence of collagen compaction resulting from expansion of CRC clusters, conceivably supported by collagen synthesis by the fibroblasts to resist invasion [21]. Further work to dissect the relative roles of overall tissue properties and localised biomechanical microenvironments in our models would be informative given the dynamic and evolving microenvironment driving cancer cell invasion [36].

TG2 is multi-functional with context-dependent activity [5], linked to several processes associated with aggressive behaviour in cancer cells including chemoresistance, resistance to apoptosis and invasive behaviour [3,6,7,9]. We observed distinct roles for TG2-derived from CRC cells and fibroblasts. In the context of fibroblasts, TG2 appeared to restrict CRC growth through collagen remodelling and matrix stiffening. However, CRC-derived TG2 appeared to drive fibroblast behaviour, and blocking CRC-derived TG2 inhibited this ability. These findings are consistent with an emerging role for TG2 in the link between cancer-fibroblast cross-talk and collagen remodelling in the ECM. TG2 secreted from pancreatic cancer cells causes activation of fibroblasts through ECM alterations [20], and TG2 secreted from breast cancer cells through microparticles is known to cause activation of fibroblasts [37]. A potential role for extracellular vesicles in mediating this tumour-driven fibroblast response is feasible—small particles released from cancer cell clusters at the invasive edge were observed in our model (Figure 1A), but confirmation that these particles are active participants in this cross-talk is necessary.

The role of LOX in mediating biomechanical changes at the tumour microenvironment is well established [19]. LOX and TG2 both have activity towards several common ECM substrates including collagen [38], although the mode and structural consequences of their activity are distinct. TG2 siRNA down-regulated LOX expression and the enzymes co-localised at the invasive edge in the co-culture model. However, this relationship was less clear in patients, and co-localisation was not clearly associated with outcome compared to TG2 alone. There is therefore rationale to investigate this relationship more closely in order to understand whether the impact of fibroblast-derived TG2 on ECM biomechanics is related to TG2 directly, to TG2-linked LOX expression, or to the interaction between these two enzymes in shaping the collagen remodelling in response to CRC. Regardless of mechanism, our data demonstrates an important role for TG2, which has an impact on disease prognosis. In vivo expression of TG2 is associated with recurrence of disease and poorer overall survival. We propose that this may be due to increased stiffening linked to collagen fibre thickening driving enhanced biomechanical signalling [17,18].

## 4. Materials and Methods

### 4.1. Cell Culture and siRNA

The primary adenocarcinoma cell line SW480 and the patient-matched cell line SW620 derived from a lymph node metastasis were obtained from the European Collection of Authenticated Cell Cultures and maintained in DMEM + 10% foetal calf serum (FCS). Primary colon fibroblasts were isolated and established from patients undergoing resection surgery at Southampton NHS University Trust as part of a prospective National Institute of Health Research study (UKCRN ID 6067), as previously described [39]. Clinical characteristics have been described previously, briefly patient donors were 1 female and 2 male Caucasian patients with stage II–III disease, aged between 68–79 [39]. Primary CAFs were obtained from the surgical area directly around the tumour, and paired normal fibroblast lines (NOF) isolated from healthy tissue at least 5 cm proximal to the tumour. In total, 3 paired fibroblast lines were obtained from 3 patient donors; 460, 463 and 602, and were isolated and maintained in DMEM + 20% FCS. All cells were cultured at 37 °C with 5% CO_2_, and used after expansion into sufficient numbers for 3D culture between passage 4 and 10. Directly before use, cells underwent treatment with either siRNA for TG2 (Stealth RNA, Life Technologies, Paisley, UK), or a negative scrambled control siRNA (control siRNA), using Lipofectamine RNAiMax (Life Technologies) as previously described [6]. Transfection was allowed to proceed for 24h before trypsinising, counting, and either plating into monolayer or into 3D culture/co-culture gels, which were supplemented with TGF-β1 (Peprotech, London, UK) where indicated. Western blotting was performed on cell lysates prepared in 1% SDS + protease inhibitors (Roche, Burgess Hill, UK) using previously described protocols [6]. Blots were probed with anti-TG2 mouse monoclonal antibody 7402 (AbCam, Cambridge, UK), anti-LOX rabbit monoclonal antibody (AbCam), anti-SMA mouse monoclonal antibody clone 1A4 (Sigma-Aldrich, Dorset, UK) and anti-actin C-11 (Santa Cruz Biotechnology, Dallas, TX, USA) primary antibodies at a dilution of 1:2000, and visualised using Li-Cor donkey secondary antibodies at 1:10,000.

### 4.2. Colorectal Cancer/Fibroblast Co-Culture

A 3D co-culture model was adapted from previously described organotypic culture protocols [22]. Briefly, the gel mixture was prepared as 57.5% collagen type I (3 mg/mL, BD Bioscience, Wokingham, UK) and 17.5% GelTrex basement membrane components (Life Technologies) in DMEM + 10% FCS. Once expanded primary colorectal fibroblasts were suspended in this mixture at 550,000 cells/mL, in the presence/absence of SW480 or SW620 cells at 275,000 cells/mL. Gels were cast at 120 μL/well in 96-well plates and allowed to set for 3 h at 37 °C forming a cylindrical shape before removal from the casting plate, and cultured untethered in 12-well plates in DMEM + 10% FCS. Medium was changed every 2–3 days for a 7-day period, prior to biomechanical testing. Once tested, gels were fixed in 10% formalin overnight, transferred into 70% ethanol and processed for paraffin embedding, sectioning, hematoxylin & eosin (H&E) staining and sirius red staining at Bart’s Pathology Service, Queen Mary, University of London. Analysis of culture supernatants was performed using commercial ELISA kits (IL-6 and IL-8, Peprotech).

### 4.3. Biomechanical Analysis

Cultures were removed from culture medium, and placed fully submerged into PBS within a square transparent perspex dish allowing visual calibration of the testing head level on the gel surface. Any defective or mis-shaped (non-cylindrical) gels were omitted from analysis. Unconfined compression tests were performed using an Instron 5967 equipped with a 10 N load cell with a resolution of 0.1 mN. Test specimens were subjected to a global strain of 30% using a flat stainless-steel platen applied at 1%/s and the resulting load was recorded. Gel height was measured using the test platen and calculated as the difference between the dish base and the top of the gel (determined using a pre-load of 0.5–1 mN). Gel diameter was measured using callipers and used to calculate gel area. Stress was calculated using the recorded force and gel area, and the modulus of each gel was calculated from the slope of the gel stress-strain curve at the linear region from 25–30% strain, termed the final modulus. Each experimental condition was cast as 3–4 gels per experiment, in a minimum of 3 independent experiments.

### 4.4. Immunofluorescence and Immunolocalisation

Immunostaining was performed on slides taken from the 3D gels processed as described above, and on tissue sections taken from patients with CRC. The Tissue microarray (TMA) used has been described previously [29], along with clinical parameters matching the samples. Briefly, TMA3 consisted of tissue biopsies obtained in triplicate from 66 patients, with equal numbers of patients who had recurrence of disease or no recurrence. These patients had a mean (±SD) age at diagnosis of 75 (±10.9, patients with recurrence) and 71.9 years (±10.5, patients with no recurrence), overall gender distribution of 49 male, 17 female patients, and similar T stage distribution between the two groups (overall T2 *n* = 4, T3 *n* = 41, T4 *n* = 22) with N stage of 0 for all patients [29]. Two slides, TMA3A and TMA3B were analysed for TG2 and LOX with DAPI as a counterstain using a protocol adapted for multi-colour fluorescence staining. Briefly, the TMAs were deparaffinised in the xylene substitute Sub-x (Leica, Milton Keynes, UK) twice for 1.5 min, then rehydrated in ethanol twice for 1.5 min. After washing in water and PBS, enzymatic antigen retrieval was performed. A 15 min incubation in 0.02% HCl at 37C was followed by 45 mins with 0.02% HCl + 2.5 mg/mL pepsin (Sigma-Aldrich), before 3 washes in PBS. Protein block solution (DAKO) was added for 20min at RT, then primary antibody solution prepared in 5% donkey serum/PBS were added overnight at 4C, using anti-TG2 mouse monoclonal antibody 7402 (AbCam) and anti-LOX rabbit monoclonal antibody (AbCam) at a dilution of 1:500 after optimisation for specific staining (Supplementary Figure S5A). After 3 PBS washes, alexa-fluor (AbCam) secondary antibody solutions were prepared in 5% donkey serum/PBS, and added for 1h at RT in the dark, using donkey anti-mouse 488, anti-rabbit 555, and anti-goat 647 at a dilution of 1:500. After a PBS wash, slides were immersed in 1% sudan black solution to dampen background autofluorescence (Supplementary Figure S5B) for 5 min at RT in the dark, before thorough washing 10 times in PBS. DAPI (Life Technologies) counter-staining was performed for 5 min at RT in the dark, and after 3 PBS washes slides were mounted in Pro-Long anti-fade (Life Technologies) and cured for a minimum of 72 h before whole-slide scanning on an Axio Scan Z1 machine (Zeiss, Cambridge, UK). Individual section images were exported from Zeiss Zen software. Sections from collagen gel cultures were stained following the same IMF protocol, with additional antibodies anti-ε-(γ-glutamyl) lysine isopeptide mouse monoclonal antibody (AbCam) and anti-SMA clone 1A4 mouse monoclonal antibody (Sigma-Aldrich) used after confirmation of specific staining using isotype controls (Abcam, Supplementary Figure S5C,D), and images captured on an inverted fluorescence microscope.

### 4.5. Image Processing and Tissue Analysis

All images were processed as multi-layer tiff files using Image-J [40]. For analysis of in vitro cultures (6 experimental runs in total), calculation of CRC cluster diameter was performed using particle analysis on H&E images. A threshold was set and particles incorporating fewer than 2 cells were excluded. A mean of 13 clusters per image were measured, with a minimum of 3 images per condition. Collagen density measurements were obtained by measuring staining intensity of selected areas from sirius red images, following background subtraction. Fibre characteristics were established using CT-FIRE software (LOCI, University of Wisconsin at Madison, MI, USA [28]) on these sirius red stains following normalisation by automated background subtraction and contrast adjustment. Data was calculated for individual fibre length, linearity (straightness), and thickness, from a mean of 138 fibres per image obtained from a minimum of 3 images per condition. For TMA analysis, staining intensity was quantified for each core by taking automated percentage values (after setting a threshold) from each channel using MaxIntensity threshold settings after rolling background correction, and co-localisation was quantified using Coloc2 analysis, with Mander’s coefficients above threshold (tM1) used as a readout. Analysis of second harmonic generation (SHG) emanating from this TMA has been previously described [30], and was re-analysed using CT-FIRE. Means of individual fibre measurements (up to 1000 per section) were compiled and used for correlation with fluorescence intensity measurements. Fluorescence values from TMA sections were quantified as mean fluorescence intensity (MFI) values, which was then normalised to tissue area using the DAPI signal. Survival analysis was performed in SPSS after splitting into two groups (high and low expression) with cut-offs at the median, and both survival analysis and comparison of expression between groups of patients were performed using mean values generated from 3 separate cores.

### 4.6. Statistical Analysis

Survival analysis was performed by Kaplan-Meier test using SPSS software (www.ibm.com/SPSS/modeller). All other statistical analyses were performed in Graphpad Prism (www.graphpad.com/scientific-software/prism) using non-parametric tests following confirmation of non-normal distribution of the data by Shapiro-Wilk test. Conditions were compared by Mann-Whitney *U* test or Kruskall-Wallis test for comparing two or more groups, respectively, and Pearson’s test was used to define correlations between variables. Significance was accepted at *p* < 0.05.

## 5. Conclusions

This study has demonstrated a multi-functional role for TG2 in the progression of CRC. Using in vitro 3D modelling, we have shown that fibroblast-derived TG2 and CRC-derived TG2 have different roles in the TME. Fibroblast-derived TG2 promotes matrix stiffening, whereas CRC-derived TG2 promotes fibroblast activation. Matrix stiffening correlates with altered collagen structure, notably increased fibre thickness. Fibre thickness and TG2 also correlate in patients with CRC, where TG2 associates with disease recurrence and poorer overall survival. This suggests that the stromal response mounted by fibroblasts to invasive CRC which initially restrains tumour growth leads to matrix alterations which contribute to poor outcome in patients.

## Supplementary Materials

The following are available online at https://www.mdpi.com/2072-6694/11/5/701/s1, Figure S1: CRC cells alter the structure of collagen fibres in a TG2-dependent manner, Figure S2: TG2 does not impact on number of SW480 clusters in co-culture with fibroblasts, but does impact on cytokine release, Figure S3: Biomechanical characterisation of co-culture gels, Figure S4: LOX expression in SW480/fibroblast co-cultures, Figure S5: Optimisation of immunofluorescent staining.

## Abbreviations

TG2	Transglutaminase-2
CRC	Colorectal cancer
NOF	Normal fibroblast
CAF	Cancer-associated fibroblast
ECM	extracellular matrix
SMA	smooth muscle actin
LOX	Lysyl oxidase
TMA	Tissue microarray
TME	Tumour microenvironment
SHG	Second harmonic generation
H&E	Hematoxylin and Eosin
