# Supplementary material for: Transglutaminase-2 Mediates the Biomechanical Properties of the Colorectal Cancer Tissue Microenvironment that Contribute to Disease Progression
Source: Cancers (Basel). 2019 May 21;11(5):701. doi: 10.3390/cancers11050701 (PMC6562428; doi:10.3390/cancers11050701)
Supplement: Supplementary file 1 [file cancers-11-00701-s001.pdf]

Supplementary Materials

Transglutaminase-2 Mediates the Biomechanical Properties of the Colorectal Cancer Tissue Microenvironment that Contribute to Disease Progression

Robin Delaine-Smith, Nicola Wright, Chris Hanley, Rebecca Hanwell, Rahul Bhome, Marc Bullock, Cole Drifka, Kevin Eliceiri, Gareth Thomas, Martin Knight, Alex Mirnezami and Nicholas Peake

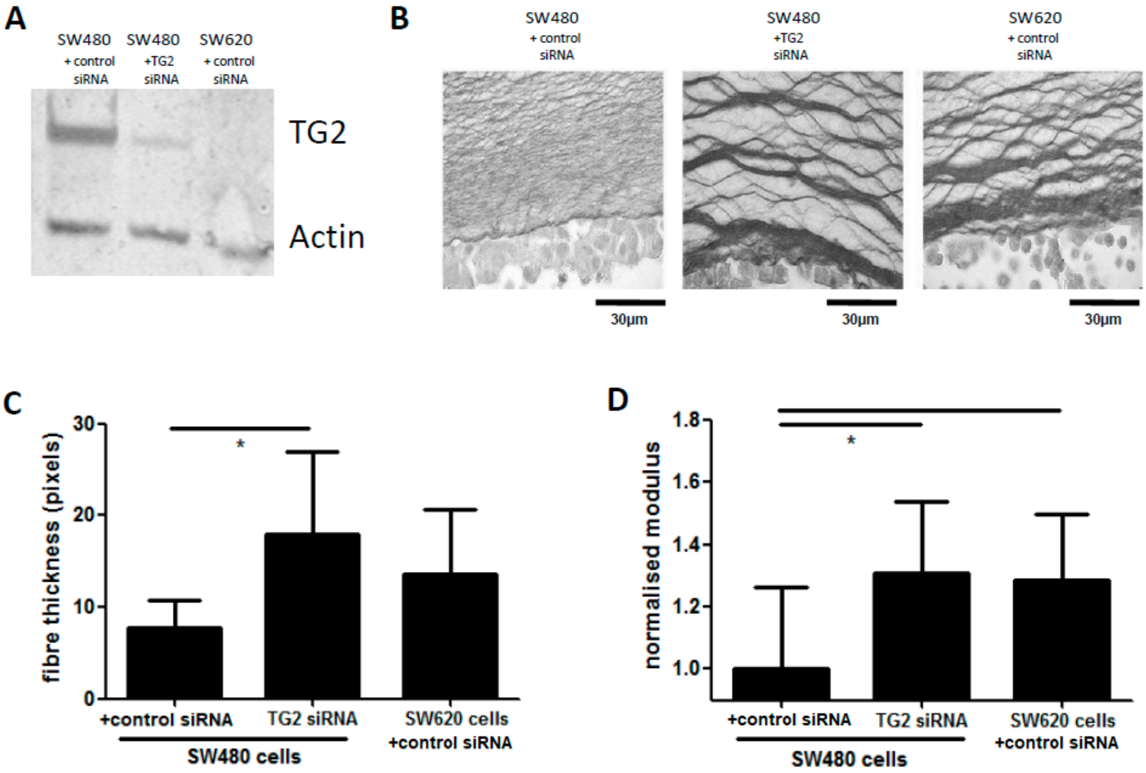

Figure S1. CRC cells alter the structure of collagen fibres in a TG2-dependent manner. SW480 cells express high amounts of full-size TG2 (**A**), which is effectively knocked down by siRNA treatment to levels similar to those of SW620 cells. Small amounts of LOXL2 were expressed in SW480 cells (**A**). To analyse the impact of the differential expression functionally, SW480 cells were applied to collagen gels and allowed to invade for 1 week (**B**, left panel). Silencing TG2 in these cells lead to a distinctive alteration in collagen fibre structure, with thickening of fibres into a more porous network (centre panel). This distinctive pattern resembled that observed when SW620 cells were applied to the gels (right panel). Quantitative analysis of these changes indicated significant alterations to collagen fibre thickness (**C**), with low TG2 associated with thicker fibres. This thickening was associated with an increased modulus value in gels treated with cells expressing low TG2 (**D**). * $p < 0.05$.

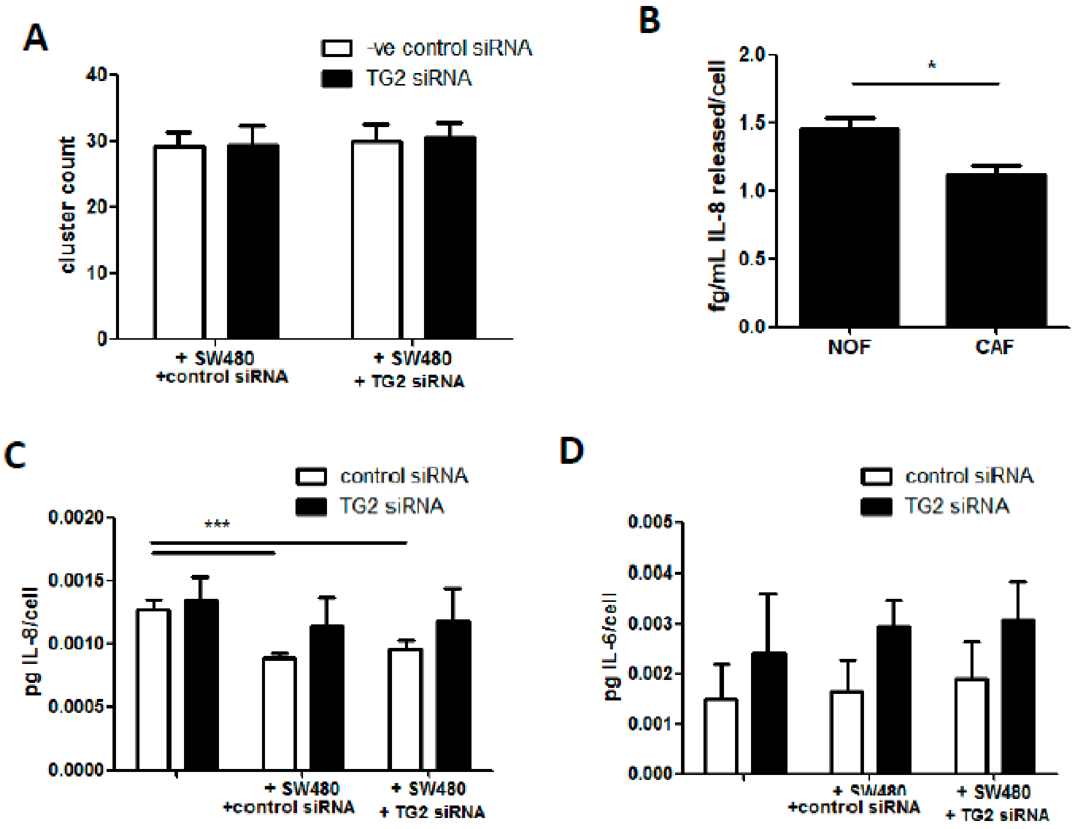

Figure S2. TG2 does not impact on number of SW480 clusters in co-culture with fibroblasts, but does impact on cytokine release. Silencing TG2 in either fibroblasts or SW480 cells did not alter the number of cancer clusters forming in the co-culture models. The role of TG2 in IL-8 release from fibroblasts was investigated using ELISA on cell culture supernatants. TG2 siRNA significantly inhibited IL-8 synthesis induced by TGF-b treatment (**A**). In the 3D gel models, IL-8 release was lower from CAFs compared to paired NOFs (**B**), and Co-culture of fibroblasts and SW480 cells significantly inhibited the release of IL-8 (**C**, white bars), however this effect was abrogated by silencing TG2 in the fibroblasts (black bars). IL-6 release from the co-cultures tended to be higher when TG2 was silenced in the fibroblasts, but this was not statistically significant (**D**), and silencing TG2 had no effect. * $p < 0.05$, *** $p < 0.001$.

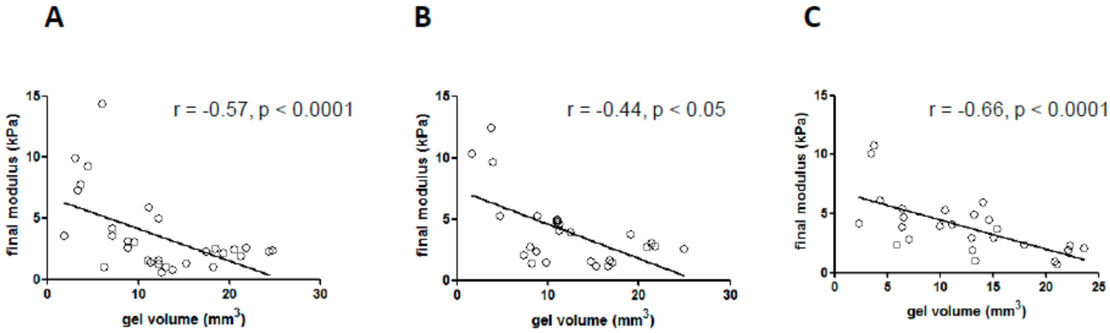

Figure S3. Biomechanical characterisation of co-culture gels. Negative correlations between modulus and gel volume, figures show experiments grouped as fibroblasts only (**A**), fibroblasts co-cultured with SW480 cells (**B**), and fibroblasts co-cultured with SW480 cells treated with TG2 siRNA (**C**).

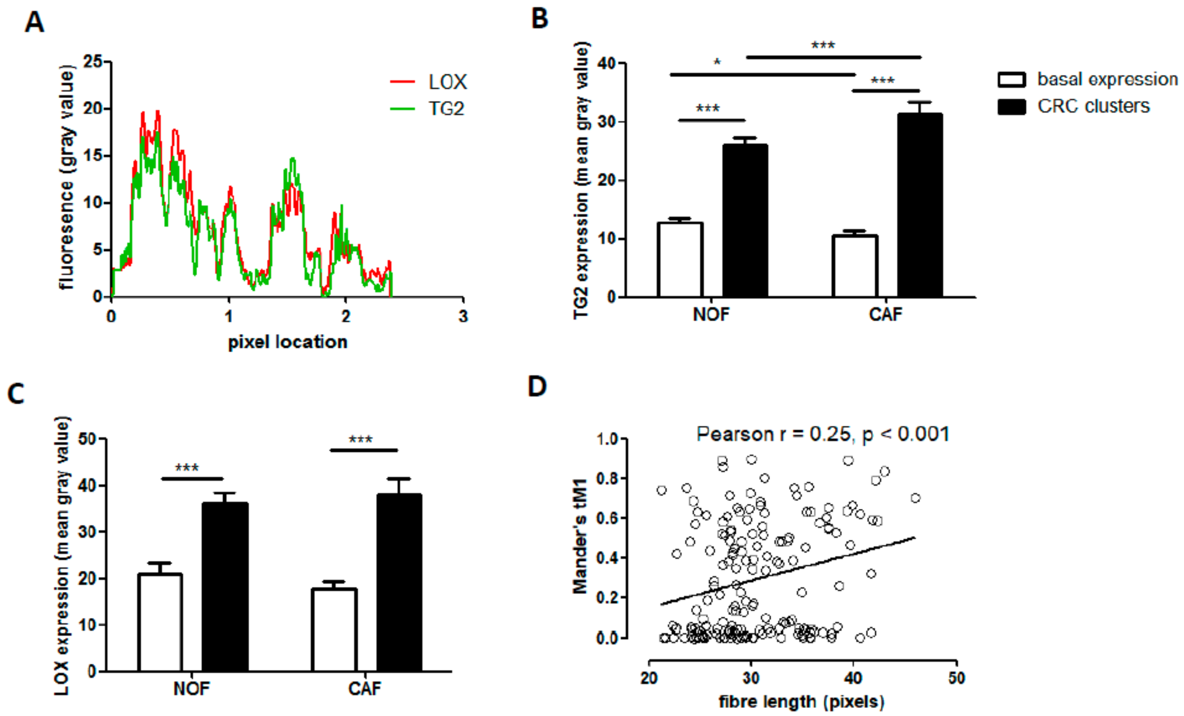

Figure S4. LOX expression in SW480/fibroblast co-cultures. Quantification of immunofluorescence intensity showed that TG2 (green) and LOX (red) signals co-localised closely (**A**). This quantification also indicated slightly less basal levels of TG2 expression in CAFs compared to NOFs (**B**, open bars), but expression at the cluster perimeter was upregulated to a greater level in CAF co-culture compared to NOFs (**B**, black bars). LOX expression was not significantly different between NOFs and CAFs but was significantly higher at the cluster perimeter (**C**, open bars are basal levels of expression, closed bars are levels at the cluster perimeter). * $p < 0.05$, *** $p < 0.001$.

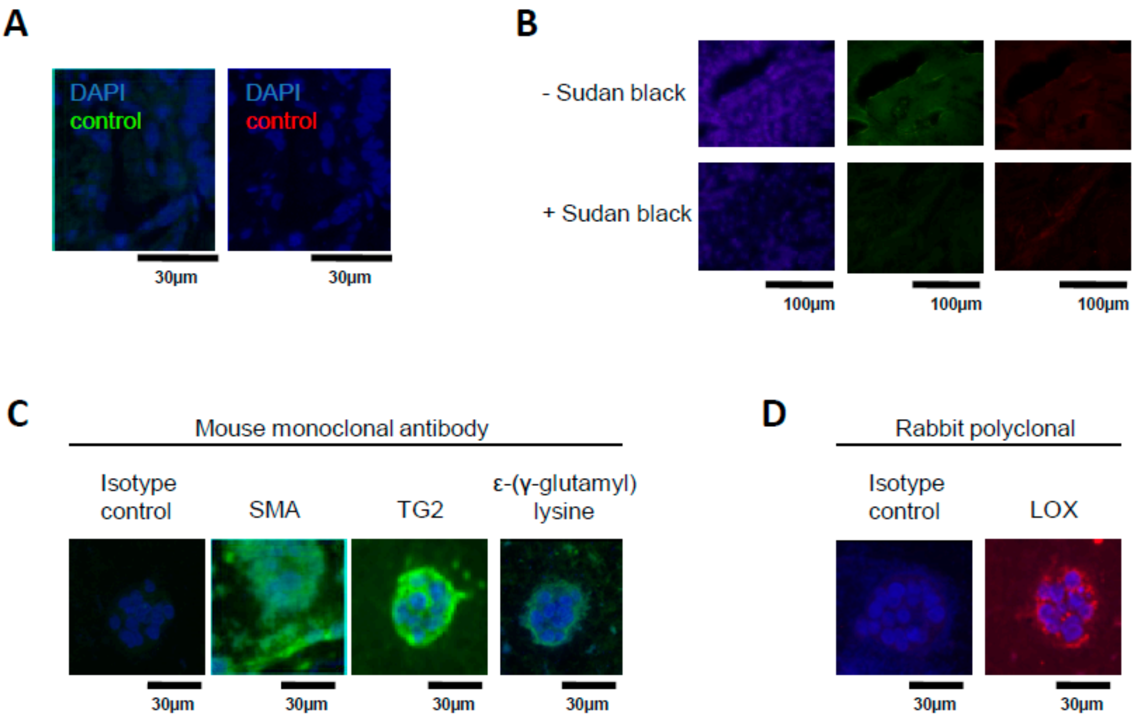

Figure 5. Optimisation of immunofluorescent staining. Low background staining was established using isotype control antibodies in tissue sections (**A**), after treatment with sudan black to dampen autofluorescence (**B**). Co-culture models were also stained using a larger panel of antibodies, which included mouse monoclonal (**C**) and rabbit polyclonal antibodies (**D**) which were tested alongside isotype control antibodies.

© 2019 by the authors. Licensee MDPI, Basel, Switzerland. This article is an open access article distributed under the terms and conditions of the Creative Commons Attribution (CC BY) license (http://creativecommons.org/licenses/by/4.0/).
